# Patients views on which return-to-work outcomes should be prioritised: A nominal group technique focus group

**DOI:** 10.1177/03080226211072766

**Published:** 2022-03-02

**Authors:** Kay Bridger, Blerina Kellezi, Denise Kendrick, Jade Kettlewell, Jain Holmes, Stephen Timmons, Isabel Andrews, Stephen Fallon, Kate Radford

**Affiliations:** 1 Division of Primary Care, School of Medicine, 6123 Nottingham University, Nottingham, UK; 2 Department of Psychology, School of Social Sciences, 6122 Nottingham Trent University, Nottingham, UK; 3 Business School, 12207 Nottingham University, Nottingham, UK; 4 Division of Rehabilitation, Aging and Wellbeing, School of Medicine, 6123 Nottingham University, Nottingham, UK

**Keywords:** Traumatic injuries, patient outcomes, vocational rehabilitation, lived experience

## Abstract

**Objective:**

Injuries can have a long-lasting effect on ability to return to work, but there is little research on which outcomes are most important to patients. This study aims to identify and prioritise return-to-work outcomes important to patients for evaluating vocational rehabilitation interventions.

**Methods:**

Nominal group technique focus group with trauma patients.

**Results:**

Focus group participants (*n* = 6) included mostly traumatic brain injuries, a range of occupation types, ages and both genders. Participants identified and prioritised their eight most important outcomes which were: sense of purpose and life satisfaction, understanding the impact of injury, assessment of readiness to return to work, using SMART (specific, measurable, achievable, relevant and time-bound) goals, facilitated reintegration to work, assessing capacity to return to work, collaboration between key stakeholders and improved employer and employee knowledge. Many of these were measures of the process of, rather than change outcomes of vocational rehabilitation.

**Conclusions:**

The range of outcomes identified by trauma patients highlights the complex process of return to work and the need for vocational rehabilitation evaluations to incorporate a broader range of outcomes. Measures of the process of vocational rehabilitation are also important to trauma patients and should be included in such evaluations.

## Introduction

Traumatic injuries are a worldwide health problem, with an estimated 56 million hospital admissions annually (Haagsma et al., 2016). For many working age adults, return to work is delayed by the injury effects, with one third of patients admitted to hospital after injury not having returned to work 12 months later (Kendrick et al., 2017). Return to work has many benefits including improved finances, self-esteem, social connection and quality of life (Waddell and Burton, 2006). Vocational rehabilitation (VR) (Escorpizo et al., 2011) interventions improve return-to-work rates for conditions including brain or spinal injuries and mental health problems (Cancelliere et al., 2016; Donker-Cools et al., 2016; Roels et al., 2016).

Generating evidence of the effectiveness of VR interventions requires consideration of which outcomes (i.e. changes brought about by the intervention or those relating to VR processes) should be measured and evaluated. However, selecting outcomes is difficult because specific work-related outcomes (e.g. work status and sickness absence) or broader outcomes (e.g. quality of life), may not capture the complexity of return-to-work processes (Wasiak et al., 2007) or the wide range of potential mediators or moderators of return to work affecting VR interventions (Saltychev et al., 2013). The choice of outcomes that capture the complexity of return-to-work processes is poorly informed by existing evidence as reviews of return-to-work following traumatic injury show absence of good quality Randomised Controlled Trial (RCT) studies and outcomes (Saltychev et al., 2013). Evidence highlights the importance of using a biopsychosocial approach to inform and evaluate VR interventions (Condon et al., 2019), which should be reflected in outcome measurement. Evidence also highlights the need to capture the heterogeneity of the lived experiences of patients, the priorities of different stakeholders (Wasiak et al., 2007) and the importance of social relations between stakeholders for return to work (Tjulin et al., 2010). Additionally, research advocates investigating further return-to-work process outcomes which capture priorities for patients (Hees et al., 2012; Roels et al., 2016; Vogel et al., 2011) that are distinct from change outcomes such as productivity, pay and time off work (Young et al., 2005). In fact, capturing the views of patients with lived experience is essential not only to inform intervention development but also the methods of evaluating its effectiveness (Skivington et al., 2021).

Our study aimed to identify and prioritise return-to-work outcomes of importance to patients (both in terms of change and processes) to inform an evaluation of a VR intervention delivered by occupational therapists and clinical psychologists to patients admitted to UK major trauma centres (Kendrick et al., 2021). The VR intervention (approved by NHS Ethics Committee) adopts a biopsychosocial approach and has been developed to work at both remedial (treating physical and psychological problems which may impact on return to work) and the social and environmental levels (adapting the work environment, job, role, responsibilities and changing the trauma survivors, co-workers’ and employers’ attitudes and confidence through education and supported self-management).

## Methods

### Research design

A nominal group technique (NGT) (Delbecq et al., 1975) focus group was used to identify and prioritise return-to-work outcomes important to trauma patients. The NGT is inclusive of all participants’ experiences, generating rich data, achieving consensus and allowing data prioritisation in a relatively short time period through the voting process.

Participants were recruited from a group of trauma patients providing patient and public involvement (PPI) input into the design and evaluation of the VR intervention (Kendrick et al., 2021). The members of the PPI group were recruited into the wider study using a variety of methods, aiming to identify a heterogeneous groups with a wide range of injury, employment, educational and socio-economic characteristics. Our PPI recruitment framework aimed for heterogeneity in occupation classification types, age, gender and type of injury to encourage diverse inputs. All PPI members were invited to take part in the NGT focus group. A time where most of those willing to take part were available, was agreed. All participants provided written consent. Participants own experience of VR support varied.

### Data collection and analysis

Participants were provided with a 15-min overview of the proposed VR intervention and detailed explanation of the NGT process. Frequent reference was made to the single question guiding the discussion: *What are the most important outcomes of a return-to-work intervention for people who have experienced traumatic injury?* The NGT stages were: (1) *Generation of Ideas*: participants silently wrote their answers to the question; (2) *Round Robin Discussion:* audio recorded anonymous reading of generated ideas, with amalgamation of similar points (3) *First Vote:* participants chose and ranked their six priority outcomes (to make second ranking more manageable); (4) *Discussion of First Vote*: the six priority outcomes were reported to the group, and the group’s discussion was audio-recorded (eight outcomes were taken to the final vote to accommodate four pairs of equally ranked items from the first vote) and (5) *Final Vote*: participants each ranked the priority outcomes in their preferred order. Group ranking was calculated by summing ranked scores from individual participants (i.e. ranked first = 6; ranked last = 1).

The data collected included individually written notes, voting cards, flip charts, moderator notes, audio recordings and an Excel spreadsheet with individual rankings.

## Results

Six trauma patients participated in the NGT focus group. Participants included those with traumatic brain injuries and musculoskeletal injuries, and a range of occupation types, ages and both genders. The participants’ injuries had occurred between three and 14 years prior to the NGT focus group. All participants had returned to work following their injury. Participant summary characteristics are presented in Table 1.

**Table 1.** Summary of nominal group technique focus group participant characteristics (*n* = 6).

Characteristic	Details
Age range	M = 51, SD = 14
	Median = 53
	Full range: 30–69 years
	IQR: 36–66
Gender	2 female
	4 male
Ethnicity	All white British
Employed/self-employed/student at time of injury	5 employed
	1 self-employed
Occupation classifications	1 each of
	Managers
	Professionals
	Technicians and associate professionals
	Craft and related trades
	Plant and machine operators
	Armed forces
Injury type	1 polytrauma + traumatic brain injury
	1 musculoskeletal upper limb injury
	4 traumatic brain injuries
Time since injury	M = 7, *SD* = 4
	Median = 6
	Full range: 3–14 years
	IQR: 5–12
Returned to work following injury	6
Now retired	2

### Top 18 outcomes identified by the participants

Eighteen discrete outcomes important to trauma patients were elicited and agreed by the participants in the first discussion phase (described and illustrated with examples in Table 2). The outcomes can be grouped into four categories, with some outcomes relevant to more than one category:

1. *Quality of life* (outcomes A: sense of purpose/work–life balance/work satisfaction/life satisfaction, and O: reintegration into society),
2. *support in return to work* (outcomes B: facilitated reintegration back to work, C: understanding impact of injury, D: assessment of readiness to return to work, E: assessment of capacity to return to work, F: improved knowledge and awareness of employer/employees, L: return to pre-injury employment, N: alternative employment and meaningful activity and Q: empowered by provision of information),
3. *ability to maintain work performance and engagement* (outcomes C: understanding impact of injury, F: improved knowledge and awareness of employer/employees, H: identifying, reviewing and achieving SMART goals, I: sustainable/appropriate work adaptations, J: confidence in ability to work, K: ongoing access to psychological and OT support, M: ability to self-manage, Q: empowered by provision of information and R: ongoing performance review)
4. *managing injury and related challenges* (outcomes C: understanding impact of injury, G: collaborative partnership between key stakeholders, K: ongoing access to psychological and OT support, N: alternative employment and meaningful activity, M: ability to self-manage, P: financial stability and Q: empowered by provision of information).

**Table 2.** Outcomes important to trauma survivors, ranked from first nominal group technique vote.

**Outcome elicited during generation of ideas phase (agreed summarisation phrase in preparation for vote) *P = participant; F = facilitator-illustrated with quotations from the discussions***	Number of participants who voted per outcome	Sum of scores per outcome (where max. score = 36)	Ranked priority in full list of 18 outcomes (= indicates joint ranking)
Participant characteristics (gender, occupational classification and injury type)			
1: Female, professional and TBI			
2: Male, manager and TBI			
3: Female, associate professional and musculoskeletal			
4: Male, armed forces and TBI			
5: Male, craft and related trade and musculoskeletal and TBI			
6: Male, plant and machine operator and TBI			
**Sense of purpose + work/life balance + work satisfaction + life satisfaction**	6	27	1
*P4: Sense of purpose is – it is a reason to do*			
*P 2: To get up in the morning*			
*P 4: It is something to live for each day*			
*P 2: Hmm. A story to tell when other people get home from work*			
P 4: You are trying to achieve your new full potential			
*P 1: To me, sense of purpose almost sounds like* the other two combined. Work and life *satisfaction give you a sense of purpose. Or life satisfaction, a sense of purpose and work satisfaction*			
B **Facilitated reintegration back to work**	4	17	= 2
P 1: To feel comfortable with colleagues on your return to work			
P 3: Not feeling that everyone is making allowances, treading on eggshells or working round you. […] Ensure work do not expect too much too quickly. Have slow and phased return. Also don’t expect too much too soon from yourself. …. I’ve got this little thing in my head that says, how are other people going to react when I come back? I don’t want there to be any sort of distance between us. So it’s about – I think there is a bit in there about the individual having a little bit of responsibility as well			
C **Understanding impact of injury**	4	17	= 2
*P 4: It is an insight, it’s an insight of the timeline, short, medium, long-term. Do you* understand the injury, do you understand the *impact if it has impacted on your life, on your work? Have you been able to mitigate against those changes? And if not, what are your goals?*			
P6: If you feel you understand your limitations			
P1: But I was glad that I was stopped. Because I would have tried to go straight back to work full-time and that would have had quite negative consequences I think			
D **Assessment of readiness to return to work**	2	11	= 3
P 4: one is if the individual is actually ready to do work, any work, is he or she ready to actually leave therapeutic support, leave the hospital, leave rehabilitation and return to work full stop? Then the other one is actually a psychological… the outcome is to determine psychologically whether the individual is ready to return to work			
P5: Then again, you know a sole trader could lie through his teeth. It’s all right saying whoever you work for, blah di blah, blah di blah, if you’re a manager. I had to have a driving licence before I could go back up a ladder. But behind your back I would have been working, couldn’t I?			
E **Assessment of capacity to return to work**	2	11	= 3
P 4: So I can’t return to work because I can’t do that, my brain won’t allow me to do that, are you meant to find something that – while I’m now, whatever my ability is. So one’s ability can change brain’s ability. And therefore that may be completely off field, a new type of work			
P 2: Hmm, I think capacity needs to be in there specifically then			
P5: Problem I had, if I couldn’t do the job I was out. So I wanted to get back see if I could do it, if that makes any sense			
F **Improved knowledge and awareness of employer/employees**	2	9	= 4
P 2: Helping the individual to self-advocate and teaching them skills for managing scenarios as they come up? Because each time I changed jobs or each time you get a promotion or a new situation you are a little bit back to square one of needing to explain to people again …[…] and kind of re-educating managers and colleagues each time […] But then if you are increasing employer awareness and understanding they’re more likely to be supportive. Possibly			
G **Collaborative partnership between key stakeholders**	2	9	= 4
P 3: It could also be part of the making sure people are financially stable. So collaborative working could be with lots of different agencies			
P 4: A lot of stakeholders are needed for that individual to return to work. […] To return to previous employment or find other suitable employment, be supported through that process. Be a partnership, listened to, supported to work			
P 2: It’s also about being heard though. Because if you were expressing things and they’re just being belittled, so the problems keep happening and get worse, that then your capability to do the job is then brought into question. When actually that’s not the reason behind it			
H **Identifying, reviewing and achieving SMART goals**	2	7	5
*P 1: when I came out of hospital I wanted to go* straight back to work pretty much or within a few months. But that wasn’t a realistic timescale because I didn’t understand the implications of my injury. So yeah, I think you need to understand the injury before you can set yourself the SMART goal			
P 4: The realistic part of SMART goals for someone who has suffered a life changing injury is understanding the implications of their injury. Once they know that they can set achievable realistic goals			
I **Sustainable/appropriate work adaptations**	4	6	
P 3: For me, one of the challenges that I found was that some of the activities that I undertook were actually more physical than they actually understood. […] So it was an element of the job role which meant that I needed to sort of rethink how it was done, find an alternative for doing it			
P 4: It’s about fitting the job to the man			
J **Confidence in ability to work**	1	5	7
P 1: To be able to highlight or discuss anxieties around my perceived performance deficits			
P3: I was fine. But there might be people out there whose self-esteem isn’t sufficient to actually get them past that and be able to talk about the anxieties they’ve got			
K **Ongoing access to psychological and OT support**	1	4	= 8
P 4: The other thing is the knowledge that if I struggle I can re-access			
P 2: And you’re empowered by knowing that you canre-access. Because I’ve been able to self-manage very early on, but then there’ll be something about when you’re the only one who’s – you’re the only voice who’s speaking you’re not always enough, in terms of other people taking it on board			
P 1: To have access to a facility for discussing how challenges make one feel			
L **Return to pre-injury employment**	2	4	= 8
P 5: Return to pre-injury employment			
P 2: But it’s far too narrow, because some people might then see that as failure if they can’t			
P 1: After a traumatic injury you often become a different person, not worse or better, a different person. And that pre-injury employment may not suit you now			
M **Ability to self-manage** P 4: The ultimate outcome is to be independent so that actually when you do want to change jobs or you want to give up work or whatever you can do it independently and you can source and find out if you need help	2	3	9
N **Alternative employment and meaningful activity**	1	1	10
P 2: Different new work using same capabilities, skills, hours but in a different job or industry. […]But meaningful activity is sometimes something towards work			
P 4: if not, can’t do that, then actually we need to then identify a new skillset with this individual because their problems are so difficult they need to start learning again and then identity a completely different type of work			
O **Reintegration into society**	0	0	—
R3: The last one was personal independence, feeling able to integrate with society			
**Facilitator**: Does sense of purpose capture the thing about sanity about returning to work or does that need to be another point?			
P6: I thought that was something to do with reintegration into society, being out of the home, out of the house			
P4: It’s about reintegrating yourself back into society I think. And not			
P6: Is it not about just avoiding boredom			
P **Financial stability**	0	0	—
P 1: I think it seems kind of obvious that that’s why people would want to go back to work, to make money, but certainly for myself and by the sounds of everyone else here, that wasn’t really a consideration when we went back to work, that’s not what we were looking for			
P5: It was [a motivation] for me.……			
*R4: Then the other one as well, there is a financial need for you to go back to work*			
Q **empowered by provision of information**	0	0	—
R4: It’s a stepping stone to being able to self-manage			
R **Ongoing performance review**	0	0	—
P 1: To be able to highlight or discuss anxieties around my perceived performance deficits			
P 3: It was the bit about getting back to actually full-time working and then looking at your next thing, your performance level and making sure that I wasn’t doing the dip. Which sometimes happens			

### The ranking order of the eight most important outcomes (Table 3)

As part of the NGT, participants ranked the six most important outcomes, although eight were retained due to equal ranking (Table 3). Columns in Table 3 indicate number of participants who voted for each outcome’s overall score and ranked position (several ranked jointly). Analysis of data from moderator notes and NGT discussions enabled further understanding of the meaning patients attributed to these eight most important outcomes. They included measures of VR processes as well as outcomes per se. Many outcomes were related to each other or were prerequisites for achieving one or more of the other outcomes.

**Table 3.** Top eight outcomes important to trauma survivors, ranked from second nominal group technique vote.

Top 8 outcomes from first vote, prioritised in second vote	Number of participants who voted per outcome	Sum of scores per outcome (where max. score = 64)
***1*** Sense of purpose + work/life balance + work satisfaction + life satisfaction	***5***	***40***
***2*** Understanding impact of injury	***6***	***38***
***3*** Assessment of readiness to return to work	***6***	***29***
***4*** Identifying, reviewing and achieving SMART goals	***5***	***24***
***5*** Facilitated reintegration back to work	***5***	***23***
***6*** Assessment of capacity to return to work	***5***	***19***
***7*** Collaborative partnership between key stakeholders	***5***	***18***
***8*** Improved knowledge and awareness of employer/employees	***6***	***16***

The most highly prioritised outcome (A) was an amalgamation of four separate outcomes: *sense of purpose/work–life balance/work satisfaction/life satisfaction*. Participants identified these outcomes in the initial-generation phase, as being sufficiently similar to group as one outcome for voting. This outcome encapsulated the importance of having a sense of purpose or meaningful work or activity (e.g. education or volunteering) which resulted in work and/or life satisfaction. This was seen to be relevant even if return to pre-injury work was not possible.

The joint second most important outcomes were *facilitated reintegration back to work* (B) and *understanding the impact of injury* (C). Outcome B referred to third-party facilitation of trauma patients’ re-joining the workplace that resulted in returners feeling ‘comfortable’ with colleagues. Outcome C encompassed the need for trauma patients, employers and colleagues to understand the short-, medium- and long-term impacts of injury on work and life in order to manage expectations about recovery by different stakeholders including patients, colleagues and employers.

The joint third most important outcomes were assessment of *readiness for* (D) and *capacity to* return to work (E). Participants clearly differentiated these two outcomes before voting, with readiness referring to processes to determine whether an individual was psychologically and physically ready to return to work. Capacity to return to work referred to the trauma patients’ ability to return to their pre-injury job and whether there was a need to identify alternative solutions.

The joint fourth most important outcomes were *improved knowledge and awareness by employers and employees* (F) regarding trauma patients’ needs and *collaborative partnership* (G) working between key stakeholders as part of the VR process. Key stakeholders included the family/close people, therapist, employer and other agencies such as the UK Department for Work and Pensions (responsible for welfare and pensions) and charities, for example, Headway.

The fifth most important outcome was *goal identification and monitoring* (H), describing the need for individually appropriate and SMART (specific, measurable, achievable, relevant and time-bound), goal setting as part of the VR process.

It is noteworthy that six of the eight most important outcomes (B, D, E, F, G and H) related to VR processes as opposed to outcomes per se.

## Discussion

### Main findings

The study’s primary aim was to identify patient priorities regarding return-to-work outcome measures to inform the effectiveness of a VR intervention. The NGT focus group identified and prioritised a range of outcomes relating to return to work of importance to trauma patients, which reflected their multiple needs and the complex process of return to work. These included outcomes focussing on the broader benefits of work for patients (e.g. providing a sense of purpose or life satisfaction; understanding the physical and psychological impact of injury) and on VR processes (e.g. assessment of readiness for and capacity to work, facilitated reintegration to work, improving knowledge and awareness of employers and employees, collaborative partnerships between stakeholders and identifying, reviewing and achieving SMART goals). Many of these outcomes were related to each other or were prerequisites for one or more other outcomes to be achieved.

### Comparison to previous research

Previous research highlights the limited and inconsistent nature of trauma outcome measurements, especially in relation to environment, activity and participation domains (Hoffman et al., 2014). Our study supports the importance of these domains, as prioritised outcomes focus on improved work environment, creating collaborative partnerships and facilitating reintegration to work. A review of return-to-work outcomes (Wasiak et al., 2007) identified goal-setting, work readiness assessment, work satisfaction, reintegration, interaction with stakeholders and appropriateness of management to be important, consistent with our study findings. Our findings also support recent qualitative research finding outcomes of importance to trauma patients include psychological and physical recovery, purposeful life engagement and managing the expectations of key stake holders (i.e. individuals, colleagues and employers) (Bridger et al., 2021). Unlike previous research, our study did not find productivity loss or return to work to be important outcomes, while highlighting the importance of measuring process as well as change outcomes (Hoffman et al., 2014).

To our knowledge this is one of the only studies of patient prioritisation of outcomes (Radford et al., 2018) for a VR intervention for survivors of traumatic injury with a range of employment, gender and age characteristics, although the majority had experienced brain injury. Evidence shows that VR intervention commissioners and research commissioners prioritise outcomes differently from patients (e.g. short-term vs longer-term) (Radford et al., 2013). Our research has highlighted that trauma patients want reassurance that the VR they receive includes some key process features that are measured, and which serve as important interim outcomes in their recovery journey.

### Strengths and limitations

Participants were recruited from a single PPI group (aiming to recruit a heterogonous membership in terms of injury, occupational and social economic characteristics) and due to unexpected cancellations, the group was smaller and had a less diverse range of injury types than planned. The number of participants was still within recommended group size for the NGT (Delbecq et al., 1975). Although our participants had experienced a range of injuries, some injuries (e.g. spinal cord injury or amputation) were not represented in our group. It is possible that other outcomes could have been identified or outcomes prioritised differently if participants with such injuries had been included. Most participants were several years into their recovery, potentially affecting recall of priorities earlier in their recovery. However, this allowed insight into medium and longer-term outcomes.

### Research implications

The diverse range of outcomes identified by patients provides important knowledge for evaluating VR interventions. For example, readiness to work and/or work self-efficacy may prove useful indicators of intervention success, especially when the dichotomous outcome of return to work is not achieved within the study follow-up period or where environmental and socio-economic factors such as economic downturns limit opportunities for return to work. Measures such as a sense of purpose or life satisfaction may be useful outcomes when return to life and work as it was pre-injury might not be possible (e.g. severe injuries) or may help explain other work outcomes. Similarly, process measures (e.g. goal setting) may be useful for quality assurance of the rehabilitation process in VR evaluations and in clinical practice. Future research should explore the role of severity and type of the injury (e.g. musculoskeletal injuries) on priority of outcomes and how outcome importance varies over time. While the present research identified sense of purpose as a key outcome, it is important to explore how this impacts on recovery and return to work.

### Practice implications

VR intervention trials should ensure they measure outcomes prioritised by trauma patients (e.g. sense of purpose and understanding injury impact), including process measures. Process measures can also be used to inform the design of VR services, quality assure services and evaluate the VR processes. Supporting people to participate in daily life including work is a primary focus in occupational therapy (Désiron et al., 2011) worldwide (WFOT 2012). Occupational therapists deliver VR in many settings worldwide and generating evidence of VR intervention is one of the key strategies of the World Federation of Occupational Therapists (WFOT, 2012). However, occupational therapists should ensure they measure outcomes that are relevant to their service-users, the intervention focus and the context in which VR is delivered.

## Key findings

• Patients from mostly traumatic brain injuries and diverse employment contexts identified key outcomes to measure in vocational rehabilitation interventions.

• Patients prioritised outcomes which include sense of purpose, life satisfaction and key stakeholder education (e.g. patients and employers).

• Patients prioritised process outcomes (e.g. assessment of readiness to return to work) as well as typical change outcomes measured in vocational rehabilitation interventions.

### What the study has added

The range of outcomes identified and prioritised by trauma patients highlights the complex process of return to work and the need for vocational rehabilitation evaluations to incorporate a broader range of outcomes. The findings also highlight the importance of involving individuals with lived experience in identifying appropriate outcome measures for a complex return-to-work intervention.

### Patient and public involvement

Patient and public involvement were involved in the study design, funding acquisition, data analysis and write up.
